# An experimental investigation of evolutionary dynamics in the Rock-Paper-Scissors game

**DOI:** 10.1038/srep08817

**Published:** 2015-03-06

**Authors:** Moshe Hoffman, Sigrid Suetens, Uri Gneezy, Martin A. Nowak

**Affiliations:** 1 Program for Evolutionary Dynamics, Harvard University, Cambridge, MA 02138, USA; 2 CentER, Department of Economics, Tilburg University, PO Box 90153, LE 5000 Tilburg, The Netherlands; 3 Rady School of Management, UC San Diego, La Jolla, 92093-0553 CA; 4 CREED, University of Amsterdam, The Netherlands; 5 Department of Mathematics, and Department of Organismic and Evolutionary Biology, Harvard University, Cambridge, MA, 02138

## Abstract

Game theory describes social behaviors in humans and other biological organisms. By far, the most powerful tool available to game theorists is the concept of a Nash Equilibrium (NE), which is motivated by perfect rationality. NE specifies a strategy for everyone, such that no one would benefit by deviating unilaterally from his/her strategy. Another powerful tool available to game theorists are evolutionary dynamics (ED). Motivated by evolutionary and learning processes, ED specify changes in strategies over time in a population, such that more successful strategies typically become more frequent. A simple game that illustrates interesting ED is the generalized Rock-Paper-Scissors (RPS) game. The RPS game extends the children's game to situations where winning or losing can matter more or less relative to tying. Here we investigate experimentally three RPS games, where the NE is always to randomize with equal probability, but the evolutionary stability of this strategy changes. Consistent with the prediction of ED we find that aggregate behavior is far away from NE when it is evolutionarily unstable. Our findings add to the growing literature that demonstrates the predictive validity of ED in large-scale incentivized laboratory experiments with human subjects.

In 1950 John Nash published a two page note in PNAS, thereby introducing what came to be known as the Nash Equilibrium (NE)^1^,^2^. Nash's result extended the boundaries of game theory to all social interactions, far beyond the two-player, zero-sum games studied by von Neumann. A historical perspective by fellow Nobel Laureate Roger Myerson proclaimed Nash's impact on the social sciences comparable to that of the double helix on the biological sciences^3^. And some 62 years post-publication, NE has become a standard tool in economics and other social sciences and the concept is so well known that it no longer needs explicit citation^4^.

Evolutionary dynamics (ED) provide an additional tool for game theorists^5^,^6^,^7^,^8^,^9^,^10^,^11^. ED specify changes in strategies over time in a population. For biological evolution, the classic ED is the replicator dynamic, where strategies reproduce at a rate proportional to their payoffs^12^. The Moran and Wright-Fisher Process are other important ED of biological evolution in finite populations^13^,^14^. These models can also be thought of as models of cultural evolution, where instead of reproducing proportional to payoffs, individuals are imitated proportional to their payoffs. Other popular ED, describing how populations of learners adjust their strategies over time, are reinforcement learning, which presumes that individuals hold more tenaciously to strategies that have performed better^15^,^16^,^17^, and stochastic fictitious play^18^. These models share the property that more successful strategies become more frequent. Typically, they also have the same stability properties^19^,^20^

The generalized Rock-Paper-Scissors (RPS) game has three strategies (Rock, Papers, or Scissors) and payoffs are such that Rock beats Scissors which beats Paper which beats Rock (Fig. 1). In this game, everyone playing rock, paper, and scissors with probability 1/3 is a NE. In this case one wins, ties and loses exactly 1/3 of the time. If everyone uses this strategy, there is no incentive to deviate. However, even though no individual can benefit by deviating, no player would be hurt by deviating either, so it is not obvious what would keep the population at this NE. This is where ED enter the story. In the RPS game, deviants would win *and* lose slightly less often than nondeviants but tie more often. Therefore, whether or not deviants outperform nondeviants, and hence whether the NE is evolutionarily stable^21^,^22^, depends on whether or not the gains from winning are larger or smaller than the loss from losing, relative to tying.

We investigate how strategies change over time in a population of human subjects playing the RPS game (Fig. 1). For all values of *a* > 1, a NE exists where each individual independently randomizes with equal probability on rock, paper, and scissors. For *a* > 2 the NE is evolutionary stable, whereas for *a* < 2 it is not. The case *a* = 2, which represents the standard children's game of RPS, is knife edge: here the deviant is neither better nor worse off, hence the NE is evolutionary stable or not depending on the ED. For instance, the NE is stable in the perturbed best-response dynamics^23^.

We ran 6 different treatments (2 feedback treatments crossed with 3 payoff treatments). For each treatment we conducted 5 sessions. In each session, 12 subjects play 100 periods of RPS, and their choice is played against the choice of all 11 other subjects. Their payoffs are evaluated and feedback is given. Rock beats scissors which beats paper which beats rock. Subjects receive 0 points for each loss, 1 point for each tie, and *a* points for a win. We ran the payoff treatments *a* = 1.1, *a* = 2 and *a* = 4. The feedback works as follows: at the end of each period, subjects learn their payoff from that round and the frequency of each strategy from that round (Frequency Feedback) or their payoff from that round and the average payoff in the group of 12 players from that round (Payoff Feedback).

We introduce the metric “distance from the center” to refer to the number of subjects, out of populations of 12, that would have to change their choice in a given period in order to get 4 rock, 4 paper, and 4 scissors. For example, if in a particular round the outcome was 8 rock, 3 paper and 1 scissors then the distance from the center of this configuration is 4. The minimum distance from the center is 0 and the maximum is 8. In total there are 
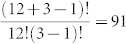
 configurations that are quantized into 9 distance levels (Fig. 2C). The distance can be averaged over all rounds of a session or treatment to become what we will refer to as a population's “average distance from the center.” The average distance from the center measures in a very intuitive way how far a population of RPS players drifts away from NE.

NE predicts the average distance from the center to be the same in all treatments (Fig. 2B). In fact, NE predicts the average distance from the center to be relatively small, although not precisely 0. Since it predicts independent randomization with equal probability on each choice, the probability of having a given distance in any given round can be calculated exactly. For instance, there is a 86.4% chance of having distance 1, 2, or 3 in any given round. And the average distance from the center for a given population/session can be calculated to be approximately normally distributed, with a mean of 1.908 and a variance of .0114, yielding a 95% chance of falling within the interval [1.701, 2.114] (see Supplementary Information).

ED, in contrast, predict a smaller average distance from the center for treatments with *a* = 4 than for treatments with *a* = 1.1. Treatments with *a* = 2 are expected to fall in between *a* = 4 and *a* = 1.1, but whether it is closer to the former or the latter depends on the precise dynamic model employed. To illustrate, refer to the phase diagrams predicted by the replicator dynamic for infinite populations shown in Fig. 3A, and to results from simulations of small populations of reinforcement learners shown in Fig. 3B.

## Results

**Figure 2:**
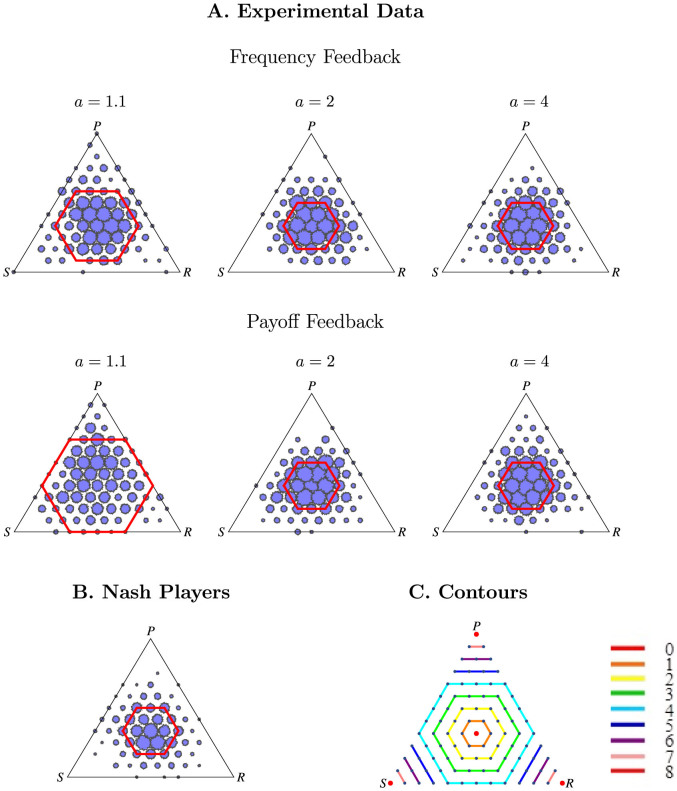
Rock-Paper-Scissors Choices in the Lab. As predicted by ED but not NE, the population distributions farther from the center occur more often when *a* = 1.1 than *a* = 2 and *a* = 4. In each of our 6 treatments, we had 5 sessions, each consisting of 12 subjects playing 100 rounds of rock paper scissors. In each round, a population distribution occurs, e.g. 8 rock, 3 paper and 1 scissors. Each population distribution can be represented by one of 91 points in an equilateral triangle, where the point in the bottom left represents the population distribution 0 rock, 0 paper, 12 scissors, and the point in the center represents the population distribution 4 rock, 4 paper, 4 scissors. Panel A shows six bubble plots over such triangles to represent the number of occurrences of each population distribution within each treatment, where the area of each bubble is proportional to the number of occurrences out of 500. The red lines connect the lattice points that are equidistant from the center and cover at least 90% of the data points. For comparison, panel B shows an additional bubble plot based on a simulation of NE, where each “individual” in each of 500 rounds independently chooses rock, paper, or scissors with equal probability. Panel C shows a plot with all possible contour lines corresponding to different distances.

Fig. 2A represents the frequency of each possible combination of rock, paper, and scissors, observed in our experiment, by treatment. As is readily apparent, the distribution is shifted away from the center in treatment *a* = 1.1 as compared to treatment *a* = 2 and treatment *a* = 4, as predicted by ED but not by NE.

Fig. 4A illustrates that this result is statistically significant; for both feedback treatments, we find evidence consistent with the dynamic prediction but not the NE prediction. Specifically, the average distance from the center is significantly larger for *a* = 1.1 than *a* = 2 and *a* = 4, according to even the most conservative tests, that is, when treating each session as a single observation, and making no parametric assumptions (p < .001 between *a* = 1.1 and *a* = 2 and p < .001 between *a* = 1.1 and *a* = 4; two-sided Mann-Whitney U tests with N = 20). In fact, we find that 9 out of 10 sessions for *a* = 1.1 fall above the 95% confidence interval constructed above. In contrast, 19 out of 20 of the sessions in *a* = 4 and *a* = 2 fall within the 95% confidence interval and 1 falls below. In the Supplementary Information, we show that these results are not sensitive to the distance metric or the non-parametric assumptions employed, nor to the type of feedback treatment.

A skeptical reader might worry that the average distance in treatment *a* = 1.1 is only so large because we haven't given our subjects enough periods to converge to NE. If this were the case, it should be expected that the average distance from the center in treatment *a* = 1.1 would be aligned with NE once we focus on periods where the population has actually hit 4 rock, 4 paper, 4 scissors. To rule out this alternative explanation, we replicate our above analysis after removing all periods that occurred in a session prior to hitting 4 rock 4 paper, 4 scissors. We find the same results (p < .001 between *a* = 1.1 and *a* = 2 and p = .002 between *a* = 1.1 and *a* = 4; two-sided Mann-Whitney U tests with N = 19). We also replicate our above analysis using only the last 50 rounds and find the same result (p < .001 between *a* = 1.1 and *a* = 2 and p = .001 between *a* = 1.1 and *a* = 4; two-sided Mann-Whitney U tests with N = 20).

A skeptical reader might also worry that our results are driven by one of the feedback treatments and won't generalize easily. Notwithstanding the observation that these two different feedback treatments induce two different dynamics, which we show below, our average distance result holds within each feedback treatment. In both feedback treatments, the average distance from the center is significantly larger in treatment *a* = 1.1 than *a* = 2 and *a* = 4 (p = .028 between *a* = 1.1 and *a* = 2, and p = .047 between *a* = 1.1 and *a* = 4 in Frequency Feedback; p = .009 between *a* = 1.1 and *a* = 2, and p = .009 between *a* = 1.1 and *a* = 4 for Payoff Feedback; N = 10 for all of the tests).

**Figure 3:**
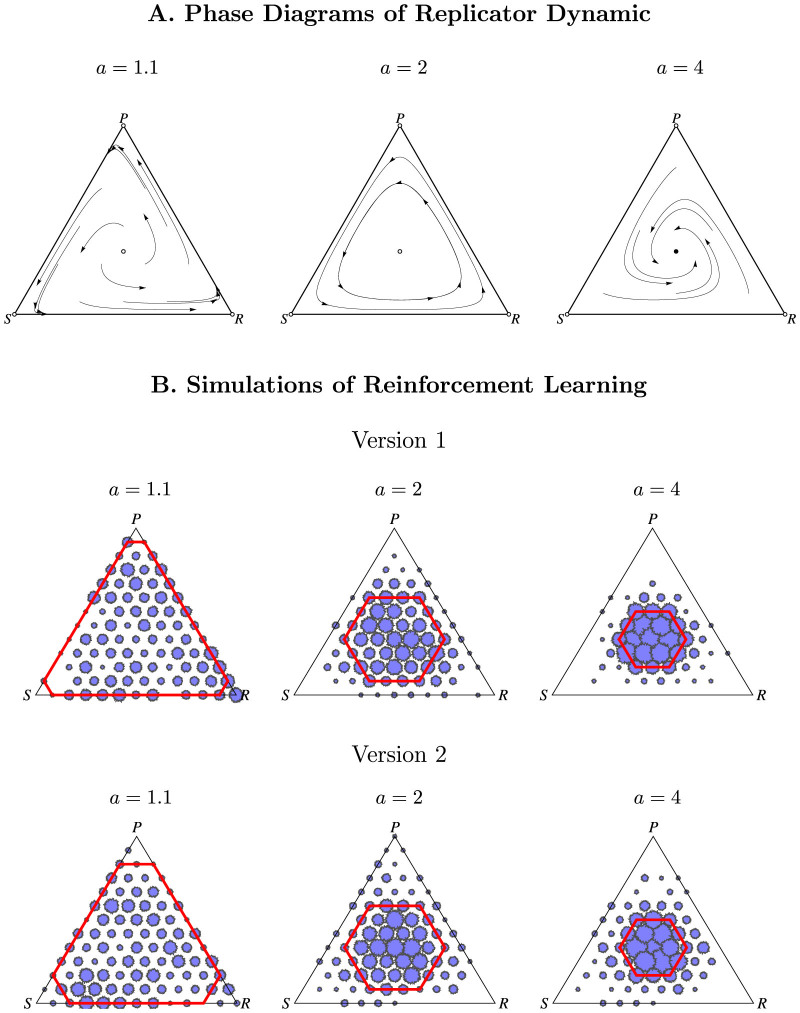
Rock-Paper-Scissors Theory and Simulations. Our main distance result is consistent with a large class of learning models. (A): In the replicator dynamic, the average distance converges toward the center when *a* = 4 (right), cycles indefinitely at a fixed distance when *a* = 2 (middle), or diverges away from the center when *a* = 1.1 (left), yielding a larger average distance in *a* = 1.1 than *a* = 4. The arrows are solutions to the ordinary differential equation^33^. (B): Stochastic simulations of reinforcement learning likewise give results similar to our experimental data; as in our experimental data, population distributions farther from the center are more frequent when *a* = 1.1 than when *a* = 2 and *a* = 4. The bubbles correspond to population distributions of rock paper scissors observed in 5 simulation runs of two versions of reinforcement learning simulations meant to follow Frequency Feedback and Payoff Feedback, respectively. The construction of this plot is identical to Figure 2 except it is based on the simulation data instead of the experimental data and is based on the same number of distinct choices (see Supplementary Information for a description of the simulation).

ED capture other important aspects of our subjects' behavior. As mentioned above, a key property predicted by ED is that individuals are expected to stay with their strategies if their strategies fare well. For instance, if a player plays rock and gets a payoff of 8 points while the average payoff for that round was only 7 points, that player is expected to be more likely to play rock in the subsequent round. This key property is most apparent in Payoff Feedback: subjects are 14.1 percentage points more likely to stay with the same strategy if one's payoff in the previous round is higher than the average payoff than if one's payoff in the previous round is lower than the average payoff (p < .001). Moreover, subjects are significantly more likely to stay with the same strategy, the higher the difference between their and the average population payoff in the previous period (marginal effect of 0.7%, p = .001). Such a learning dynamic makes sense, because the only information subjects have in Payoff Feedback, is how their payoff compares to the population payoff. What is more, such a dynamic gives rise to counterclockwise cycles (see, for example, Fig. 3A, for a smooth version of such dynamics), for which we likewise find support in the experiment. In particular, in Payoff Feedback, the number of subjects in a population choosing rock (paper) [scissors] in period *t* is positively correlated with the number of subjects in the population choosing scissors (rock) [paper] in period *t* – 1 for *a* = 1.1, 2, or 4 (p < .050). See the Supplementary Information for details of the statistical analyses.

In Frequency Feedback, the key property of ED is also evident, albeit less clearly. To see this, we need to first adjust the analysis to account for the different information available to subjects. Subjects now know the distribution of choices in the population of the previous round and not just their own choice. Consequently, it seems natural to presume that subjects are taking into account this distribution, or, at least, the modal choice, when choosing rock, paper, or scissors. Therefore, we presume that they choose between the following options: best responding to the most frequent choice of the previous period, best responding to the best response of the most frequent choice of the previous period, or best responding to the best response to the best response (i.e., mimicking the most frequent strategy of the previous period). We again check if subjects are more likely to choose one of these strategies depending on how well that strategy has fared. However, we make one alteration; since subjects cannot easily calculate the average payoff, we assume that subjects decide how well their strategy is faring by comparing their current payoff to their past payoffs. If we make these two adjustments, we obtain similar results for Frequency Feedback as for Payoff Feedback: overall, subjects are 3.2 percentage points more likely to stay with the same (higher-level) strategy if their payoff in the previous period went up (or did not change) than when it went down (p = .001). The consequence is that also in Frequency Feedback, population strategies are correlated with previous-period strategies in the predicted way. In particular, higher-level strategy cycles are counterclockwise in the sense that the population moves from many subjects playing best-response to most frequent choice to best-response to best-response to most frequent choice to mimic most frequent choice (p < .050 for *a* = 2, 4, not significant for *a* = 1.1). Details of the statistical analyses are in the Supplementary Information.

**Figure 4:**
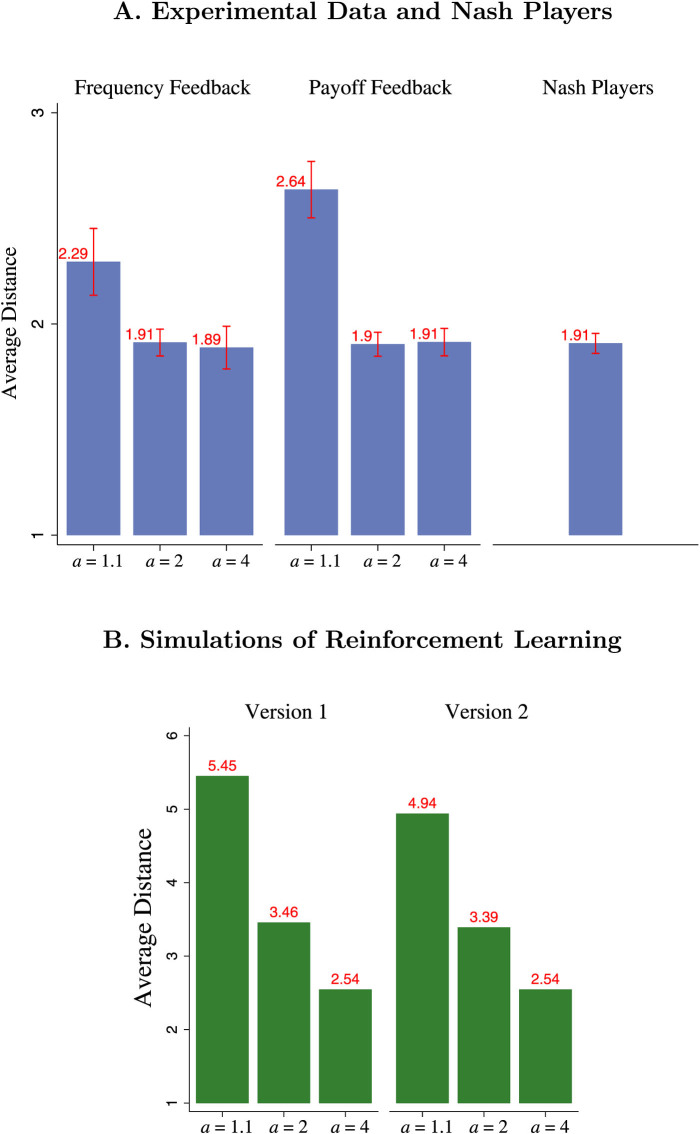
Distance from 4 Rock, 4 Paper, 4 Scissors. As predicted by ED but not by NE, the average distance is larger when *a* = 1.1 than *a* = 2 and *a* = 4. (A): The 6 leftmost bars show the average distance observed in the experiment by treatment (error bars indicate ± 1 standard error). For comparison, the right most bar indicates the average distance and standard error expected according to NE. The distance from the center for a given period of a given session is defined as the number of subjects out of 12, who would have to switch strategies to have 4 rock, 4 paper, and 4 scissors. (B): Stochastic simulations of reinforcement learning give comparative static results similar to our experimental data. As in our experimental data, population distributions farther from the center are more frequent when *a* = 1.1 than when *a* = 2 and *a* = 4. The data correspond to population distributions of rock paper scissors observed in 5 simulation runs of two versions of reinforcement learning simulations meant to follow Frequency Feedback and Payoff Feedback, respectively (see Supplementary Information for a description of the simulation).

Finally, we turn to simulations of in-game learning to reproduce our main distance result in various ED. The aim of these simulations is not to maximize the fit with the experimental data, but rather to illustrate that the distance result can be reproduced with non-deterministic ED models. Inspired by the dynamics in each feedback treatment described above, we simulated two versions of reinforcement learning models and find that population distributions farther from the center are more frequent when *a* = 1.1, and *a* = 2 than when *a* = 4 (Fig. 3B and 4B). We model Payoff Feedback using a modified version of standard reinforcement learning^16^. In the model (version 2 in Fig. 3B and 4B) the higher the difference between a player's payoff of a particular choice and the average payoff of all players in the previous period, the more likely that player is to repeat this choice in the future. For instance, if a player plays rock and gets a payoff of 8 points while the average payoff for that round was only 7 points, that player will be more likely to play rock in subsequent periods. This setup seems reasonable for Payoff Feedback, since, there, subjects do not have information about the frequency of each choice from the previous round so it would be hard to form beliefs over the frequency of each choice coming up, but subjects can form beliefs about how well their choice is faring on average. In contrast, for Frequency Feedback, it seems more reasonable to assume that subjects are updating their choices using a variant of the standard reinforcement learning model (version 1 in Fig. 3B and 4B). Instead of choosing between rock, paper, or scissors, we describe subjects as choosing between the following options: best responding to the most frequent choice of the previous period, best responding to the best response of the most frequent choice of the previous period, or best responding to the best response to the best response (i.e., mimicking the most frequent strategy of the previous period). We assume, that subjects are more likely to choose one of these strategies depending on how well that strategy has fared^23^. However, we make one alteration; since subjects cannot easily calculate the average payoff, we assume that subjects decide how well their strategy is faring by comparing their current payoff to their past payoffs. A detailed description of both dynamic models is in the Supplementary Information.

One might wonder why our subjects behave similarly in *a* = 2 and *a* = 4. Recall, that the NE is stable for some ED, such as the perturbed best-response dynamics^24^. Moreover, in our computer simulations, we observe that as *a* increases monotonically the average distance decreases, with a large difference for smaller *a*, but less of a difference for larger *a*, (see Supplementary Information) which is consistent with our finding of *a* = 1.1 being very different from *a* = 2 but *a* = 2 being indistinguishable from *a* = 4.

## Discussion

We used a simple laboratory experiment of the Rock-Paper-Scissors game to demonstrate the value of Evolutionary Dynamics (ED). We found that, as predicted by ED, when the Nash Equilibrium (NE) was not stable the population frequencies were farther from NE than when the NE was stable. This result complements a growing literature demonstrating the empirical validity of ED (e.g. Ref. 25).

Refs. 26 and 27 found similar evidence. They ran laboratory experiments of games similar to the RPS game where the NE either was or was not stable. They provide converging evidence that aggregate behavior is closer to NE when the NE is stable. Moreover, in their experiments ED yield an additional prediction different from NE: in their game, the time average of the ED is predicted to differ from NE, and they find evidence supporting this prediction. Additionally, Ref. 27 run a continuous version of their game, where players can update their strategies in continuous time. In this case, they find cycles similar to those observed in Fig. 3A. In addition to comparing stable and unstable NE, other papers have showed that ED fit time trends of behavior better than NE^10^,^16^, that changing a parameter that does not change the NE but does change ED effects laboratory behavior as predicted by ED^28^,^29^, that in Cournot games, where the NE is typically unstable^30^, behavior is more variable in Bertrand games, where the NE is stable^31^, and that cycles are often observed in pricing games^32^.

Our paper, alongside this growing literature, suggests that while NE may be a valuable predictor of behavior when the NE is stable and when there is an opportunity for learning or evolution, when the NE is not stable, it ought to be less trusted. Thus while NE provides a useful approximation, ED, and particularly stability criteria, ought to be investigated.

## Methods

The experiment was run in the lab of the Rady School of Management at UCSD with undergraduate students. The experiment had 3 × 2 treatments (*a* = 1.1, 2, vs. 4; Frequency Feedback vs. Payoff Feedback). For each of the 6 treatments, we ran 5 sessions consisting of 12 subjects each, giving 360 subjects in total. No subject participated in more than one session. Each session took about 45 minutes and average earnings were $12.4.

Subjects were randomly assigned to cubicles. Each cubicle contained a computer screen, which was only visible to the subject seated in that cubicle. Once seated, the experimenter handed out the instructions and read them out loud. The instructions explained the game and stated the payoff matrix as well as the type of feedback that will be given after each round for Subjects were then prompted to follow their computer screen (see the Supplementary Materials for a sample of the instructions and screenshots). In each period, subjects first chose between rock, paper and scissors. They then waited until all others made their choice. They then received feedback. After viewing their feedback, the next round began.

Payoffs were determined as follows: rock beats scissors, which beats paper, which beats rock. Subjects received 0 points for each loss, 1 point for each tie, and *a* points for each win, where *a* = 1.1, 2, or 4, depending on the treatment. All payoffs were rounded to the nearest decimal point. The feedback worked as follows: At the end of each period, subjects learned their own payoff from that round and the frequency of each strategy from that round (Frequency Feedback) or their payoff from that round and the average payoff in the group of 12 players from that round (Payoff Feedback).

After 100 such periods, subjects were paid in private, based on the points they earned during the experiment, with 100 points equaling $1.

The methods were carried out in accordance with the approved guidelines. All experimental protocols were approved by the IRB committee of the University of California, San Diego Human Research Protections Program. Informed consent was obtained from all subjects.

## Author Contributions

M.H., S.S. and U.G. designed the laboratory experiment. S.S. prepared the statistics and figures. M.H. and M.N. designed and performed the simulations of in-game learning. U.G. funded the laboratory experiment. M.H., S.S. and M.N. wrote the main manuscript text. All authors reviewed the manuscript.

## Supplementary Material

Supplementary Information Supplementary Info

## Figures and Tables

**Figure 1:**
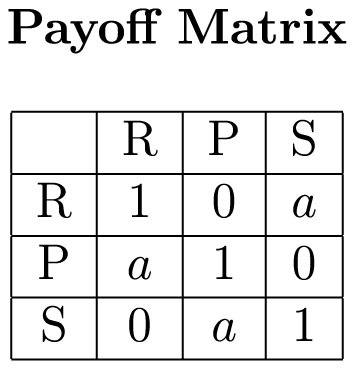
The Rock-Paper-Scissors Game. The generalized Rock-Paper-Scissors game (with *a* > 1) is a game where each player chooses between rock, paper, and scissors as in the children's game. When *a* = 2, we have the same incentives as in the children's game, but when *a* > 2 the payoff of winning is relatively high compared to the payoff of tying, and when *a* < 2 the payoff of winning is relatively low compared to the payoff of tying.
