# Supplementary material for: An experimental investigation of evolutionary dynamics in the Rock-Paper-Scissors game
Source: Sci Rep. 2015 Mar 6;5:8817. doi: 10.1038/srep08817 (PMC4351537; doi:10.1038/srep08817)
Supplement: Supplementary Information — Supplementary Info [file srep08817-s1.pdf]

An experimental investigation of evolutionary dynamics in the Rock-Paper-Scissors game

Moshe Hoffman, Sigrid Suetens, Uri Gneezy, and Martin A. Nowak

Supplementary Information

---

1 Methods and procedures
- 1.1 Experimental procedures
- 1.2 Sample instructions
- 1.3 Screenshots

2 Supporting analyses
- 2.1 Distance calculations under NE
- 2.2 Robustness of main experimental distance result
  - 2.2.1 Various distance metrics
  - 2.2.2 Distance from 4 rock, 4 paper, 4 scissors by session
  - 2.2.3 Parametric tests
  - 2.2.4 On insufficient adjustment time
  - 2.2.5 Distance from 4 rock, 4 paper, 4 scissors by feedback treatment
- 2.3 Dynamics
  - 2.3.1 Dynamics within experimental sessions
  - 2.3.2 Win-stay lose-shift
  - 2.3.3 Monotonicity
  - 2.3.4 Population autocorrelation

3 Simulations
- 3.1 Simulation models
- 3.2 Simulation results

Tables S1 to S4, Figures S1 to S9, and references

1 Methods and procedures

1.1 Experimental procedures

360 undergraduate students from UCSD were recruited from a preexisting subject pool and flyers on campus. Subjects were told that they would play Rock-Paper-Scissors for around 45 minutes and would make around $12, depending on their performance. For each of the 6 treatments, we ran 5 sessions consisting of 12 subjects. No subject participated in more than one session. Each session took about 45 minutes and average earnings were $12.4. The experiment was conducted using z-Tree—a computer program designed to run laboratory games (1).

In each session, subjects showed up in groups of 12 and were randomly assigned to cubicles. Each cubicle contained a computer screen, which was only visible to the subject seated in that cubicle. Once seated, the experimenter handed out the instructions and read them out loud. The instructions explained the game and stated the payoff matrix as well as the type of feedback that will be given after each round (see Section S1.2 for the instructions for one of the treatments). Subjects were then prompted to follow their computer screen (see Section S1.3 for screenshots). In each period, subjects first chose between rock, paper and scissors. They then waited until all others made their choice. They then received feedback. After viewing their feedback, the next round began.

Payoffs were determined as follows: rock beats scissors, which beats paper, which beats rock. Subjects received 0 points for each loss, 1 point for each tie, and a points for each win, where a = 1.1, 2, or 4, depending on the treatment. All payoffs were rounded to the nearest decimal point. The feedback worked as follows: At the end of each period, subjects learned their own payoff from that round and the frequency of each strategy from that round (Frequency Feedback) or their payoff from that round and the average payoff in the group of 12 players from that round (Payoff Feedback).

After 100 such periods, subjects were paid in private, based on the points they earned during the experiment, with 100 points equaling $1.

1.2 Sample Instructions (*a* = 1.1, Payoff Feedback)

In this experiment you will be asked to make a number of decisions. Each participant receives the same instructions. Please follow the instructions carefully. At the end of the experiment, you will be paid your earnings in private and in cash.

Please do not communicate with other participants. If you have a question, please raise your hand and one of us will help you.

During the experiment your earnings will be expressed in points. You start with no points. Points will be converted to dollars at the following rate: 100 points = $1.

The experiment is anonymous: that is, your identity will not be revealed to others and the identity of others will not be revealed to you.

Specific Rules

In the experiment you will play the game of Rock-Paper-Scissors 100 times.

In the Rock-Paper-Scissors game you either choose Rock, Paper, or Scissors. The rule is that Rock beats Scissors, Scissors beats Paper, and Paper beats Rock. See the Figure below [figure was included to illustrate the Rock-Paper-Scissors game].

In the experiment, you win 1.1 point each time you beat an opponent, you win 0 points each time an opponent beats you, and you win 1 point each time you choose the same strategy as an opponent.

During each of the 100 rounds of play, you play against all 11 other participants in this room. That is, your choice will be played against the choices of all other participants.

After each round you will learn the average payoff made by all participants in the round, and your payoff for the round.

Do you have any questions?

1.3 Screenshots

Choice entry screen in all treatments:

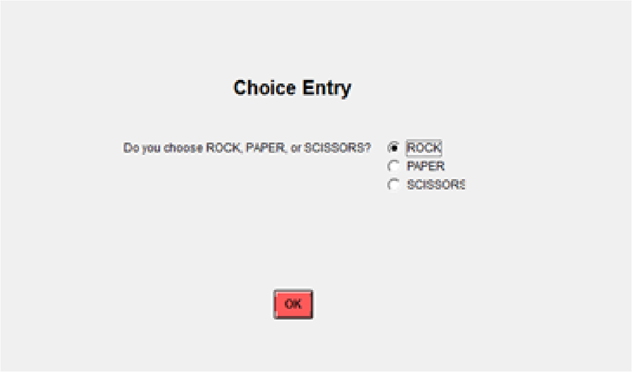

Information screen in Frequency Feedback:

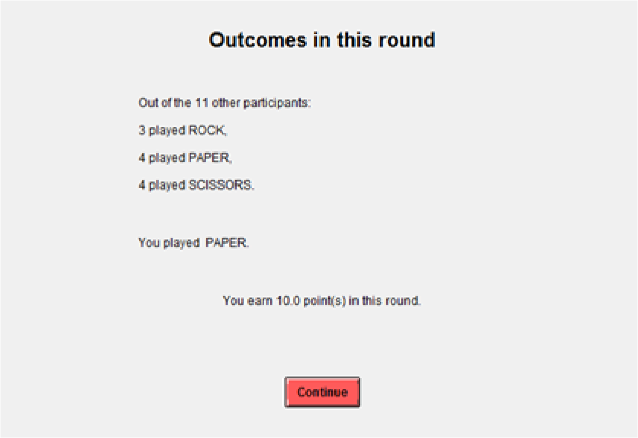

Information screen in Payoff Feedback:

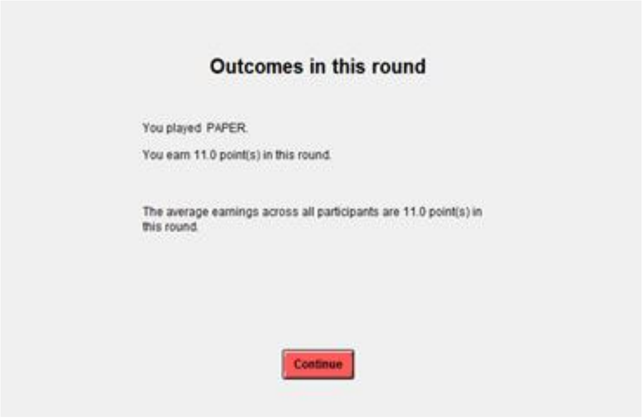

2 Supporting analyses

2.1 Distance calculations under NE

According to NE, in each round, each of the twelve players independently chooses rock, paper, or scissors with equal probability. The probability of each of the $\frac{(12+3-1)!}{12!(3-1)!} = 91$ configurations can, therefore, be calculated using the probability mass function for the multinomial distribution, with 12 draws and three equally likely categories. For instance, 4 rock, 3 paper, and 5 scissors has probability .052.

We define the *L1* distance norm as $\frac{|\sum \text{rock} - 4| + |\sum \text{paper} - 4| + |\sum \text{scissors} - 4|}{2}$. This is the metric used in the manuscript and can be interpreted as the number of subjects that would need to switch strategies in order to reach the population configuration of 4 rock, 4 paper, 4 scissors. Each of the 91 configurations corresponds to one of 9 possible *L1* distances. For instance, 4 rock, 3 paper, and 5 scissors has *L1* distance 1, since one individual would have to switch from scissors to paper to yield a 4 of each.

The probability of each *L1* distance can therefore be calculated by summing the probabilities of each of the configurations that yield that distance. For instance, the probability of obtaining distance 1 is .31296 since there are 6 configurations of *L1* distance 1 and each have probability .052. The probability that a configuration of *L1* distance 2 and distance 3 is hit in any given round can likewise be calculated to be .355 and .197 respectively, yielding a probability .864 of having distance 1, 2 or 3.

The expected *L1* distance and variance in *L1* distance per round can be calculated based on the probabilities of each distance, yielding an expected *L1* distance of 1.908 and a variance in *L1* distance of 1.114.

According to NE, each round is also independent of the previous round. Hence, the average *L1* distance in a session is approximately normally distributed with mean 1.908 and variance .114, by the central limit theorem. This distribution has a 95% confidence interval of [1.701, 2.114]. The 95% confidence interval for all 500 rounds in a given treatment is approximately [1.815, 2.000].

We can likewise define the *L2* distance metric as $\sqrt{\frac{(\sum \text{rock} - 4)^2 + (\sum \text{paper} - 4)^2 + (\sum \text{scissors} - 4)^2}{4}}$. There is no natural interpretation for this metric. And we can calculate the 95% CI over 100 rounds in a given session is approximately [1.112, 1.380] and the 95% CI over 500 rounds in a given treatment is [1.193, 1.309].

Finally, we define the *likelihood* distance metric as follows: the *likelihood* metric for a given round is simply the likelihood of obtaining the observed population configuration under NE, i.e., under the multinomial distribution described above. The *likelihood* metric for a given session is simply the average likelihood over all rounds in that session. This metric has the natural interpretation that sessions with higher *likelihood* are more likely to occur under NE.

We can compare this *likelihood* metric to that expected under NE. Under NE, the *likelihood*

metric can be calculated to have a CI of [.0301, .0374] when obtained using 100 rounds, and [.0321,.0354] when obtained using 500 rounds, based on the same method we use for *L1*.

2.2 Robustness of main experimental distance result

Herein we show our main finding that the average distance is larger in treatment a = 1.1 than in treatments a = 2 and a = 4 is not due to the distance metric employed, non-parametric assumptions, insufficient adjustment time, outlier sessions, or type of feedback provided.

		Frequency Feedback			Payoff Feedback		
		L1	*L2*	*likelihood*	*L1*	*L2*	*likelihood*
a = 1.1	1	2.16*	1.409*	.0292*	2.48*	1.628*	.0246*
	2	1.99	1.299	.0327	2.39*	1.548*	.0255*
	3	2.28*	1.474*	.0289*	3.06*	1.964*	.0171*
	4	2.9*	1.869*	.0215*	2.41*	1.558*	.0267*
	5	2.14*	1.409*	.0300*	2.84*	1.817*	.0198*
	All	2.294*	1.492*	.0285*	2.636*	1.703*	.0227*
a = 2	1	2.04	1.351	.0313	1.93	1.265	.0333
	2	2.07	1.342	.0308	2.11	1.372	.0305
	3	1.87	1.237	.0341	1.84	1.223	.0348
	4	1.85	1.230	.03.7	1.86	1.225	.0343
	5	1.73	1.141	.0370	1.78	1.169	.0352
	All	1.912	1.260	.0334	1.904	1.250	.0337
a = 4	1	2.06	1.351	.0308	2.09	1.365	.0310
	2	2.06	1.341	.0312	2	1.309	.0323
	3	1.78	1.168	.0359	1.85	1.210	.0356
	4	2	1.304	.0325	1.92	1.254	.0337
	5	1.54*	1.013*	.0410*	1.71	1.126	.0367
	All	1.888	1.235	.0343	1.914	1.253	.0339

Table S1. Various Distance Metrics by Treatment and Session. The table gives an overview of averages by session and across sessions by treatment of the *L1* distance norm, the *L2* distance norm, and the *likelihood* distance norm. Stars indicate averages fall outside respective CI.

2.2.1 Various distance metrics

According to all 3 of our distance metrics described in Section 2.1 (*L1*, *L2*, and *likelihood*), the average distance in a given treatment fall above the 95% CI for a = 1.1 but not a = 4 and a = 2, as displayed in the rows marked “all” of Table S1.

Also, in the main text we show *L1* is significantly larger for a = 1.1 than a = 2 and a = 4, according to Mann-Whitney U tests treating each session as an independent observation. The

same qualitative result is obtained for *L2* and *likelihood*. Specifically, for both *L2* and *likelihood* $p < .001$ between $a = 1.1$ and $a = 2$ and $p < .001$ between $a = 1.1$ and $a = 4$ (two-sided Mann-Whitney U tests with N = 20).

2.2.2 Distance from 4 rock, 4 paper, 4 scissors by session

The remaining rows of Table S1 display the average distance for each session according to all 3 of our distance metrics described in Section 2.1. Using all 3 distance metrics, 9 out of 10 sessions for $a = 1.1$ fall above the 95% confidence interval under the null assumption of NE, constructed in Section 2.1, while 19 out of 20 of the sessions in $a = 4$ and $a = 2$ fall within this 95% confidence interval and 1 falls below.

2.2.3 Parametric tests

In the main text we reported that the average distance from the center is significantly larger for $a = 1.1$ than $a = 2$ and $a = 4$, according to two-sided Mann-Whitney U tests. The same result holds using the parametric t-test. Particularly, $p < .001$ between $a = 1.1$ and $a = 2$ and $p < .001$ between $a = 1.1$ and $a = 4$ (two-sided t-tests with unequal variances with N = 20).

2.2.4 On insufficient adjustment time

As can be seen in Fig. S1, the treatment effect replicates when solely looking at periods after 4 rock, 4 paper, 4 scissors have been reached ($p < .001$ between $a = 1.1$ and $a = 2$ and $p = .002$ between $a = 1.1$ and $a = 4$; two-sided Mann-Whitney U tests with N = 19 in both cases). The two $a = 1.1$ treatments fall outside the 95% CI under NE, but the remaining 4 treatments do not.

Providing further evidence that our results are not due to insufficient adjustment time we turn to the last 50 periods of each session. Again, as shown in Fig. S2, we replicate our main results and find the same treatment effects in the last 50 periods ($p < .001$ between $a = 1.1$ and $a = 2$ and $p = .001$ between $a = 1.1$ and $a = 4$; two-sided Mann-Whitney U tests with N = 20). We also find that the two $a = 1.1$ treatments fall outside the 95% CI under NE, and the remaining 4 treatments do not.

2.2.5 Distance from 4 rock, 4 paper, 4 scissors by feedback treatment

Our main result holds true within each feedback treatment. In both feedback treatments the average distance from the center is significantly larger in treatment $a = 1.1$ than $a = 2$ and $a = 4$, according to two-sided Mann-Whitney U tests ($p = .028$ between $a = 1.1$ and $a = 2$ for Frequency Feedback; $p = .009$ between $a = 1.1$ and $a = 2$ for Payoff Feedback; $p = .047$ between $a = 1.1$ and $a = 4$ for Frequency Feedback; $p = .009$ between $a = 1.1$ and $a = 4$ for Payoff Feedback; N = 10 for all of the tests). Thus, our result is not just driven by one of the feedback treatments; rather, the two feedback treatments provide independent replications.

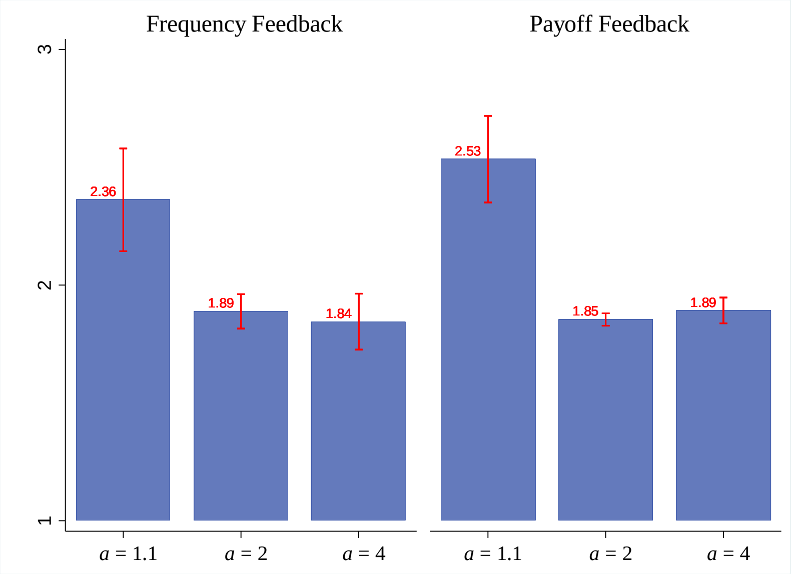

Figure S1. Distance after hitting 4 Rock, 4 Paper, 4 Scissors. The figure shows the average distance (*L1*) by treatment in periods after which 4 rock, 4 paper, 4 scissors has been reached. The CI is produced separately for each bar, based on the number of observations in that bar, e.g. for *a* = 1.1 there are 431 observations so the CI is [1.81, 2.01], and 2.36 falls above this CI.

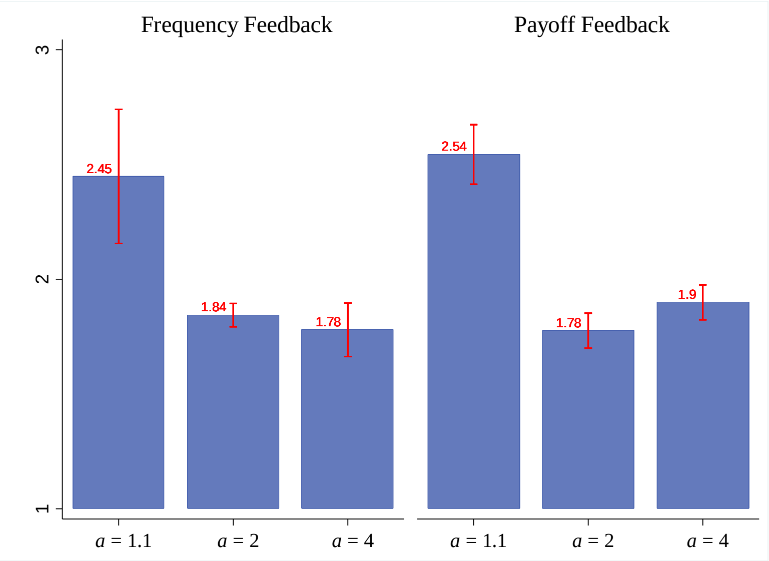

Figure S2. Distance in last 50 Periods. The figure shows the average distance (*L1*) by treatment in the last 50 periods. Treatments where a = 1.1 fall outside of the 95% confidence interval under independent randomization [1.77, 2.03].

2.3 Dynamics

2.3.1 Dynamics within experimental sessions

To illustrate how population configurations evolve over time in the lab, we include links to videos showing the evolution of configurations in the simplex, the evolution of population frequencies of rock and paper, and the evolution of distance from 4 rock, 4 paper, 4 scissors for a sample of experimental sessions. We include an experimental sessions of a = 1.1 in Payoff Feedback (link), and an experimental session of a = 4 in Payoff Feedback (link). For comparison, we also include a link to a video of a simulation of how Nash players would play, where in each of 100 periods 12 "individuals" independently choose between rock, paper, and scissors with equal probability (link). In these videos, it is readily seen that the population frequencies in a = 4 (and Nash Players) hover around the center, whereas the population frequencies in a = 1.1 seem to wander all over the place. Notice also that the population frequencies in a = 1.1 occasionally foray into the center, but immediately spring back toward the edges, whereas in a = 4 (and Nash Players) the population occasionally digresses toward the edges, but immediately gets pulled back towards the center. For convenience, Fig. S3 presents snapshots from these videos.

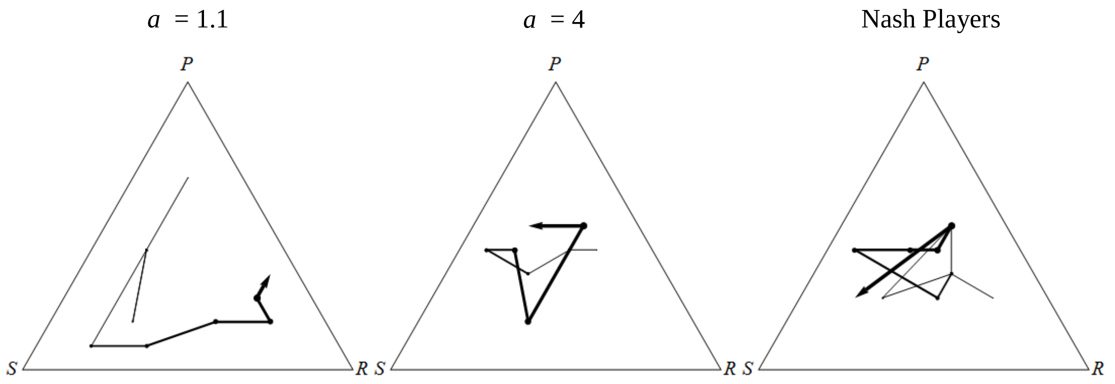

Figure S3. Population Configurations Evolving over 10 Periods. The figure shows population configurations evolving over 10 consecutive periods towards the end of 2 experimental sessions in Payoff Feedback (one session for a = 1.1 and one session for a = 4) and for Nash players.

For convenience, we also include figures showing how behavior in the lab evolves over time. Fig. S4 shows 5-period moving averages of population frequencies of rock and paper evolving over time, within each session (scissors can be inferred). Fig. S5 shows 5-period moving averages of distance evolving over time, within each session and, for comparison, simulations of 5 NE sessions, where in each of 100 rounds of each session 12 individuals independently choose between rock, paper, and scissors with equal probability. As can be seen in these figures, in particular in Payoff Feedback, observed population frequencies move around quite dramatically, as does average distance. The figures provide further evidence that our results are not due to insufficient adjustment time.

Frequency Feedback

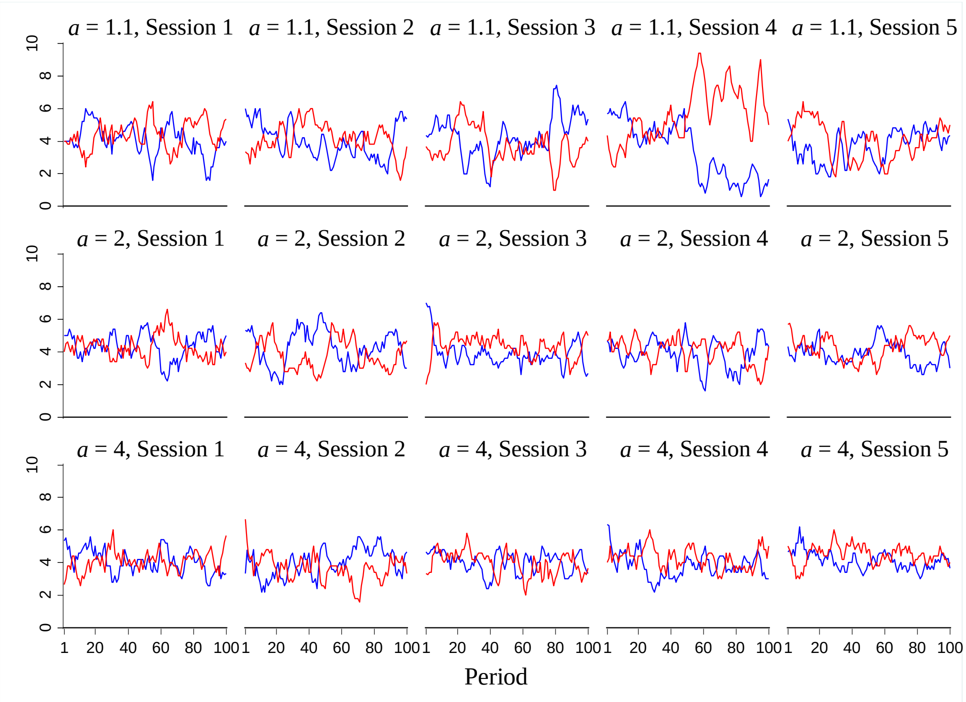

Payoff Feedback

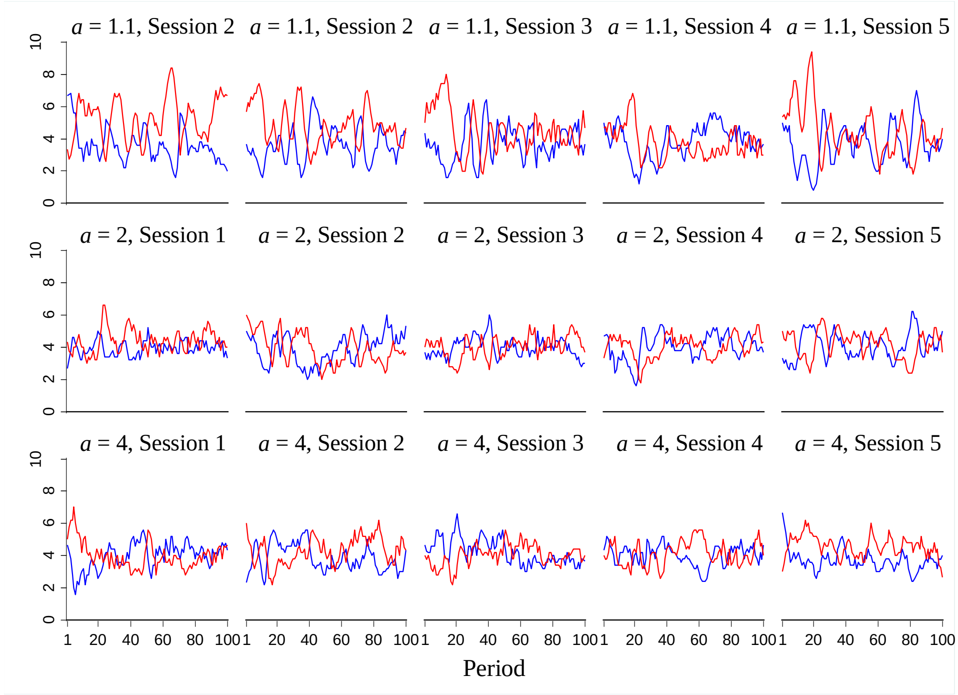

Figure S4. Population Frequencies of Rock and Paper Evolving over Time. The figure shows 5-period moving averages of population frequencies of rock (blue) and paper (red) evolving over time in the experiment.

A. Experimental Data

Frequency Feedback

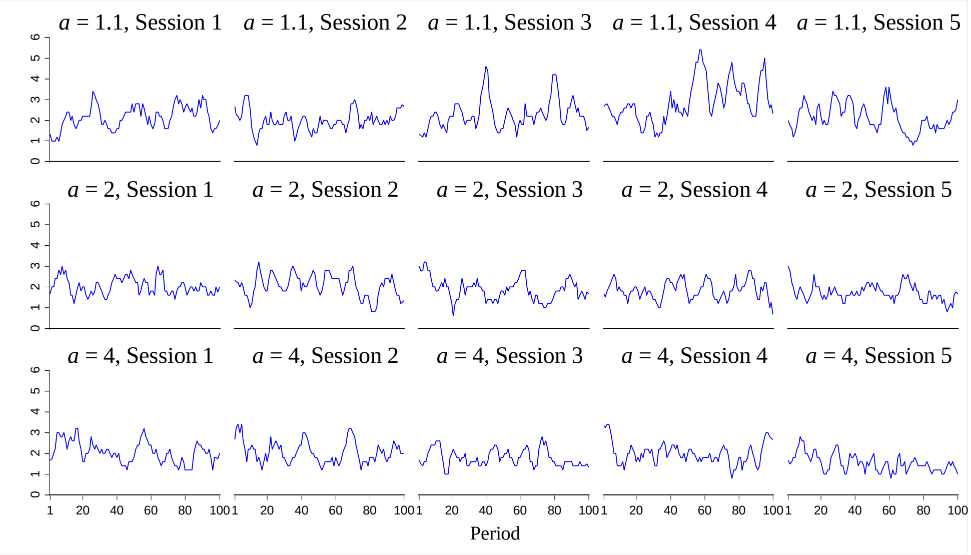

Payoff Feedback

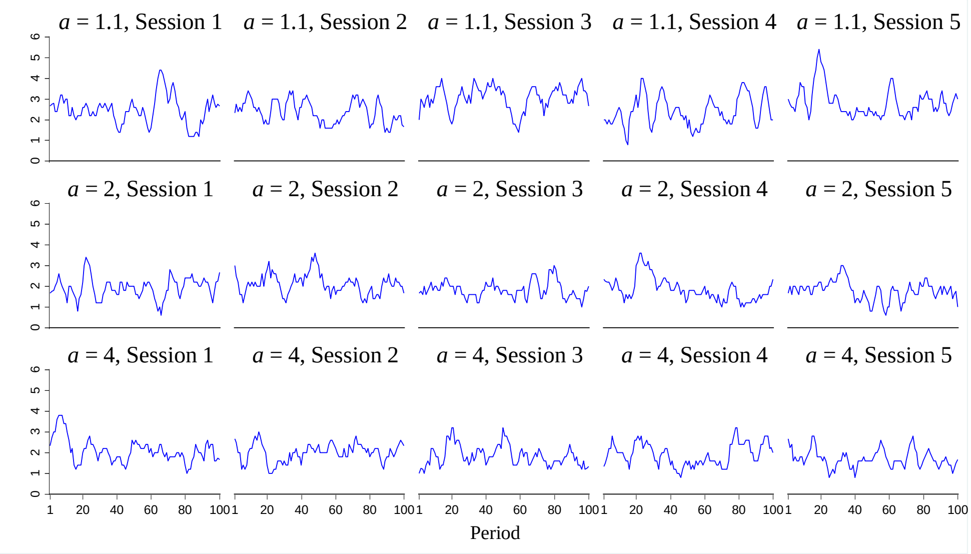

B. Simulated Nash Players

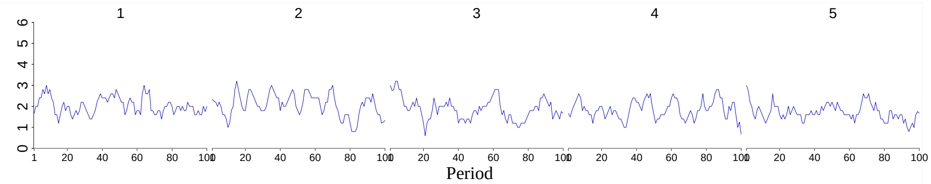

Figure S5. Distance Evolving over Time. Panel A shows 5-period moving averages of distance evolving over time in the experiment by treatment and session. Panel B shows 5-period moving averages of distance evolving over time for 5 sets of 12 Nash players.

2.3.2 Win-stay lose-shift

To demonstrate that the dynamics in the lab are characterized by win-stay lose-shift, we estimate, for each feedback treatment, the probability of staying with the same strategy in *t* as a function of whether one's payoff is higher than (or equal to) the average payoff in $t-1$. In particular, we run probit regressions where the dependent variable is a binary variable that measures whether a subject stays with the same strategy in period *t*. The independent variable is a binary variable indicating whether one's payoff in period $t-1$ is higher than (or equal to) the average payoff in period $t-1$. Standard errors are adjusted for clustering within sessions.

We find that in Frequency Feedback subjects are, overall, not more likely to stay with the same strategy if one's payoff in the previous round is higher than the average payoff than if one's payoff in the previous round is lower than the average payoff ($p = .341$). In Payoff Feedback, however, subjects are 14.1% more likely to stay with the same strategy if one's payoff in the previous round is higher than the average payoff than if one's payoff in the previous round is lower than the average payoff ($p < .001$). Results for each payoff treatment separately are shown under row (1) in Table S2.

	Frequency Feedback			Payoff Feedback		
	$a = 1.1$	$a = 2$	$a = 4$	$a = 1.1$	$a = 2$	$a = 4$
(1)	.10***	- .02	-.04	.23***	.15***	.05**
Nr. of obs	6000	6000	6000	6000	6000	6000
(2)	.02	.04**	.04***	.06***	.03	.03
Nr. of obs	5171	5100	4920	5256	4956	4896

Table S2. Win-stay lose-shift. The table gives an overview of estimated marginal effects in probit regressions. In both regressions the dependent variable is a binary variable indicating whether a subject stays with the same strategy in *t*. In regression 1 the strategy is defined as rock, paper, or scissors and in regression 2 as best-responding to the most frequent choice of the previous period, best-responding to the best response of the most frequent choice of the previous period, or best-responding to the best response to the best response (i.e. mimic the most frequent strategy of the previous period). The independent variable in regression 1 is a binary variable indicating whether one's payoff in period $t-1$ is higher than (or equal to) the average payoff in period $t-1$, and in regression 2 it is a binary variable indicating whether one's payoff in period $t-1$ is higher than one's payoff in period $t-2$. Standard errors are adjusted for clustering within sessions. Stars *** (**) [*] indicate the effect is statistically significant at the 1% (5%) [10%] level.

Providing information on own payoff and average population payoff as in treatment Payoff Feedback induces subjects to adopt a form of reinforcement learning: successful strategies—strategies with above average payoff—are "reinforced". This type of win-stay lose-shift does not show up in Frequency Feedback, at least not when the winning payoff differs substantially from the tying payoff (for $a = 2$ and $a = 4$), where information is provided about

previous-period frequencies of rock, paper, and scissors in the population instead of average payoff.

If we redefine the dependent and independent variable taking into account the different nature of feedback subjects get, we see evidence of another basic form of reinforcement learning in Frequency Feedback. If we take subjects as either best-responding to the most frequent choice of the previous period, best-responding to the best response of the most frequent choice of the previous period, or best-responding to the best response to the best response (i.e., mimic the most frequent strategy of the previous period), then we see that subjects are 3% more likely to switch to another strategy if their payoff in the previous period went down than when it went up compared to two periods prior (probit regression with robust standard errors, $p = .001$). Table S2 shows under row (2) regression results for each payoff treatment separately.

These results indicate that in both treatments subjects use a win-stay lose-shift strategy at least to some extent.

2.3.3 Monotonicity

To demonstrate that the dynamics in the lab are characterized by monotonicity, which is a crucial element of many learning and evolutionary dynamics, we estimate the probability playing rock (paper) [scissors] in period t as a function of the difference in period $t - 1$ between the payoff of rock (paper) [scissors] and the average payoff across all strategies. In particular, we run probit regressions where the dependent variable is a binary variable that measures whether rock (paper) [scissors] is chosen in period t. The independent variable is a variable equal to the payoff rock (paper) [scissors] in period $t - 1$ minus the average payoff in period $t - 1$.

We find that in Payoff Feedback the overall estimated marginal effect in this regression is equal to .0077 and statistically significant ($p = .001$), and so are the effects for each payoff treatment separately (see row (1) in Table S3). In Frequency Feedback the estimated effect is positive and significant for $a = 1.1$ but not so for $a = 2$ and $a = 4$, as also shown under row (1) in Table S3.

In Frequency Feedback, if we define the dependent and independent variable taking into account the different nature of feedback subjects get—instead of rock (paper) [scissors] we consider best-responding (best-responding to the best response) [mimicking the most frequent choice of the previous period], and past success is taken as the change in the payoff of strategy x in period $t - 1$ as compared to period $t - 2$—the data also show some evidence of monotonicity. As shown under row (2) in Table S3, marginal effects are positive for $a = 2$ and $a = 4$. Across both $a = 2$ and $a = 4$, the effect turns out as (marginally) statistically significant (equal to .00072 and $p = .056$).

Summarizing, the data show strong support for monotonicity under Payoff Feedback. Under Frequency Feedback, where the nature of feedback is entirely different, the strength and type of monotonicity seems to depend on payoffs. If winning and tying payoffs are not very

different (a = 1.1), “standard” monotonicity is observed as well. If the payoff from winning is much higher than the payoff from tying (a = 2 and a = 4), (weak) monotonicity is observed for “higher-level” strategies, that is, strategies defined in terms of steps of best-responding to the most frequent strategy of the previous period.

	Frequency Feedback			Payoff Feedback		
	a = 1.1	a = 2	a = 4	a = 1.1	a = 2	a = 4
(1)	.026***	- .003	-.004	.040***	.013***	.002**
Nr. of obs	17412	17712	17640	17424	17688	17700
(2)	.000	.001	.001	.001	.001	-.001
Nr. of obs	10656	11136	9972	11484	10020	9816

Table S3. Monotonicity. The table gives an overview of estimated marginal effects in probit regressions. In regressions 1, the dependent variable is a binary variable indicating whether a subject has chosen rock (paper) [scissors] in t. In regression 2, the dependent variable is a binary variable indicating whether a subject has chosen best-responding (best-responding to the best response) [mimicking the most frequent choice of the previous period] in t. The independent variable in regression 1 is equal to the payoff of rock (paper) [scissors] in period $t - 1$ minus the average payoff in period $t - 1$, and in regression 2 it is the change in the payoff of best-responding (best-responding to the best response) [mimicking the most frequent choice of the previous period] in period $t - 1$ as compared to period $t - 2$. Standard errors are adjusted for clustering within sessions. Stars *** (**) [*] indicate the effect is statistically significant at the 1% (5%) [10%] level.

2.3.4 Population autocorrelation

To show that the population distribution of rock, paper, scissors, observed in period t is correlated with the population distribution of rock, paper, scissors, observed in period $t - 1$, for each treatment we run a linear regression with standard errors clustered at the session level. The dependent variable is the number of subjects in a session of 12 players playing rock (paper) [scissors] in period t. The independent variables are the number of subjects playing rock (paper) [scissors] in period $t - 1$ and the number of subjects playing scissors (rock) [paper] in period $t - 1$. The upper part of Table S4 gives an overview of the regression results (under (1)).

The table shows that under Payoff Feedback, the number of subjects in a population choosing rock (paper) [scissors] in period t is positively correlated with the number of subjects in the population choosing scissors (rock) [paper] in period $t - 1$ for a = 1.1, 2, or 4. Dynamics are thus counterclockwise in the sense that the population moves from many subjects playing rock to many playing paper to many playing scissors. Such counterclockwise cycles are exactly what the replicator dynamic and related learning dynamics such as reinforcement learning would predict.

	Frequency Feedback			Payoff Feedback		
	a = 1.1	*a* = 2	*a* = 4	*a* = 1.1	*a* = 2	*a* = 4
(1)						
# strategy x in *t* – 1	.43**	.00	-.12	.53***	.26**	.07**
# strategy y in *t* – 1	.24**	-.07	-.22	.41***	.31***	.12**
R^2	.14	.01	.04	.23	.08	.01
Nr. of obs.	1485	1485	1485	1485	1485	1485
(2)						
# strategy x in *t* – 1	.22	.08*	.10**	.33***	.12**	.01
# strategy y in *t* – 1	.04	.10**	.14**	.08**	.06	.01
R^2	.04	.01	.02	.10	.001	.00
Nr. of obs.	1095	1086	1008	1152	1020	1005

Table S4. Population autocorrelation. In regression (1), the dependent variable the population frequency of rock (paper) [scissors] in period *t* , and as independent variables the population frequencies rock (paper) [scissors] in period *t* – 1 and the population frequency of s scissors (rock) [paper] in period *t* – 1. In regression (2), the dependent variable the population frequency of best-responding (best-responding to the best response) [mimicking the most frequent choice of the previous period] in period *t* , and as independent variables the population frequencies of best-responding (best-responding to the best response) [mimicking the most frequent choice of the previous period] in period *t* – 1 and the population frequency of mimicking the most frequent choice of the previous period (best responding) [best-responding to the best response] in period *t* – 1. Standard errors are adjusted for clustering within sessions. Stars *** (**) [*] indicate the effect is statistically significant at the 1% (5%) [10%] level.

Under Frequency Feedback the direction of the dynamic depends on *a*. For *a* = 1.1 dynamics are counterclockwise, whereas for *a* = 2, and particularly *a* = 4, they are rather clockwise. We suspect that subjects in Frequency Feedback try to predict the current frequency of each strategy on the basis of the observed past frequency distribution, and then best-respond to these beliefs.

Interestingly, if we redefine strategies in Frequency Feedback taking into account the different nature of feedback, i.e., in terms of the above-defined “higher-level” strategies, we see evidence of counterclockwise cycles in the sense that the population moves from many subjects playing best-response to most frequent choice to best-response to best-response to most frequent choice to mimic most frequent choice. Table S4 gives an overview of these estimation results in the lower part of the table (under (2)).

3 Simulations

3.1 Simulation models

RLF2 exponential: 12 “individuals” play 100 rounds of RPS. Before the first round, each individual *i* is endowed with a propensity for each strategy *k* in rock, paper, or scissors, denoted $\text{Propensity}_{i,k}$. $\text{Propensity}_{i,k}$ is randomly drawn from the uniform distribution between 0 and *I*, where *I* is a parameter that measures the strength of priors.

For all rounds $t = 1$ to 100, the likelihood that *i* chooses strategy *k* is proportional to e^(w*$\text{Propensity}_{i,k}$), where *w* is a parameter that represents the strength of learning.

After all players' actions are stochastically chosen, payoffs are determined for each player and their propensities update. If player *i* played strategy *k* then $\text{Propensity}_{i,k}$ increases by her payoffs minus the average over all players’ payoffs in round *t*. Propensities for all strategies other than *k* are not changed.

RLF1 exponential: The same as above except strategies are defined differently and the rule for incrementing propensities is defined differently.

Strategies are defined as follows: After each round *t* the modal choice for round *t* is determined. The strategies are no longer rock, paper, or scissors but are the modal strategy from the previous round, the best response to the modal strategy, and the best response to the best response to the modal strategy. E.g. if in round *t*, 5 scissors, 3 rock, and 3 paper were chosen, then the first strategy would dictate scissors, the second would dictate rock, and the third would dictate paper. In the case where there are two modal choices we randomly selected one of the two modes.

Propensities were incremented as follows: if player *i* played strategy *k* in period *t* then $\text{Propensity}_{i,k}$ would increase by *i*'s payoffs minus her average payoffs from periods 1 through $t - 1$. Since propensities can only increase if we know the payoffs from at least one previous period, we do not increment after the first period.

RLF2 and RLF1 non-exponential: Like above, except the probability of choosing a strategy is directly proportional to propensities instead of to likelihoods. Hence, we no longer have a *w* parameter. However, now we need to worry about negative propensities. Hence we introduce a parameter *c*, which represents the lowest amount we let propensities to be. Larger *c* indicates the extent to which individuals continue to experiment with a strategy regardless of how bad it's fared; hence a lower *c* can also be interpreted as stronger learning.

RLF1 STM: Same as RLF1 exponential above, except, we let propensities update based on the most recent move instead of just the average of all previous moves.

The above 5 simulation models are adaptations of reinforcement learning (3).

WF: Once again, 12 “individuals” play 100 rounds. In round 1, each player independently chooses a strategy with equal probability.

In each round *t*, each player's choice is played against every other player to determine their payoffs. Fitness is then calculated as 1-W+W*payoffs, where *w* is a parameter that measures the strength of selection or learning. Every player dies and a new player is born.

For each new player, with probability *u*, a parameter that represents the degree of experimentation or mutation, they choose a strategy at random. With probability 1-*u*, they mimic the strategy of one of the players from the previous generation, with probability proportional to their fitness. We did not simulate this model, but instead solved analytically for steady state.

This simulation model is an adaptation of the Wright-Fisher model (4, 5).

3.2 Simulation results

Fig. 3B and Fig. 4B in the main text are based on data for RLF1 exponential (reinforcement learning version 1) and RLF2 exponential (reinforcement learning version 2), based on 5 runs of w = .05, I = 10, and C = 1. In Fig. S7, we present the corresponding bubble plots for the remaining simulations, excluding WF, since for WF, we solved for steady state distributions instead of running finite number of times, since analytic results could be obtained. These figures illustrate that the results presented in the main text generalize to other simulations.

In Fig. S8, we demonstrate that the main distance result holds true for a large parameter region, for each of our 6 simulation models. The line corresponding to a = 1.1 is consistently above a = 2 which is consistently above a = 4, except where the three lines converge. Note that for each simulation, there is a parameter region in which there is no effect of a on average distance, likely because the parameters prevent effective learning or evolution from occurring. But never does the effect reverse; i.e. average distance is always greater for a = 1.1 than for a = 4 or there is no difference. Unlike the bubble plots, these figures and the subsequent figures were created based on 100,000 simulation runs to remove noise, since we are no longer trying to compare with experimental results. For WF the figures are based on steady state frequencies, since analytic results could be obtained.

In Fig. S9, we demonstrate that, in all 6 of our models, as a increases from 1.1 to 4 the average distance increases, albeit at a decreasing rate, possibly explaining why in our experiment our a = 1.1 treatment looks quite distinct from our a = 2 treatment, but our a = 2 treatment looks similar to our a = 4 treatment. Simulation data was obtained for values of a in increments of .1.

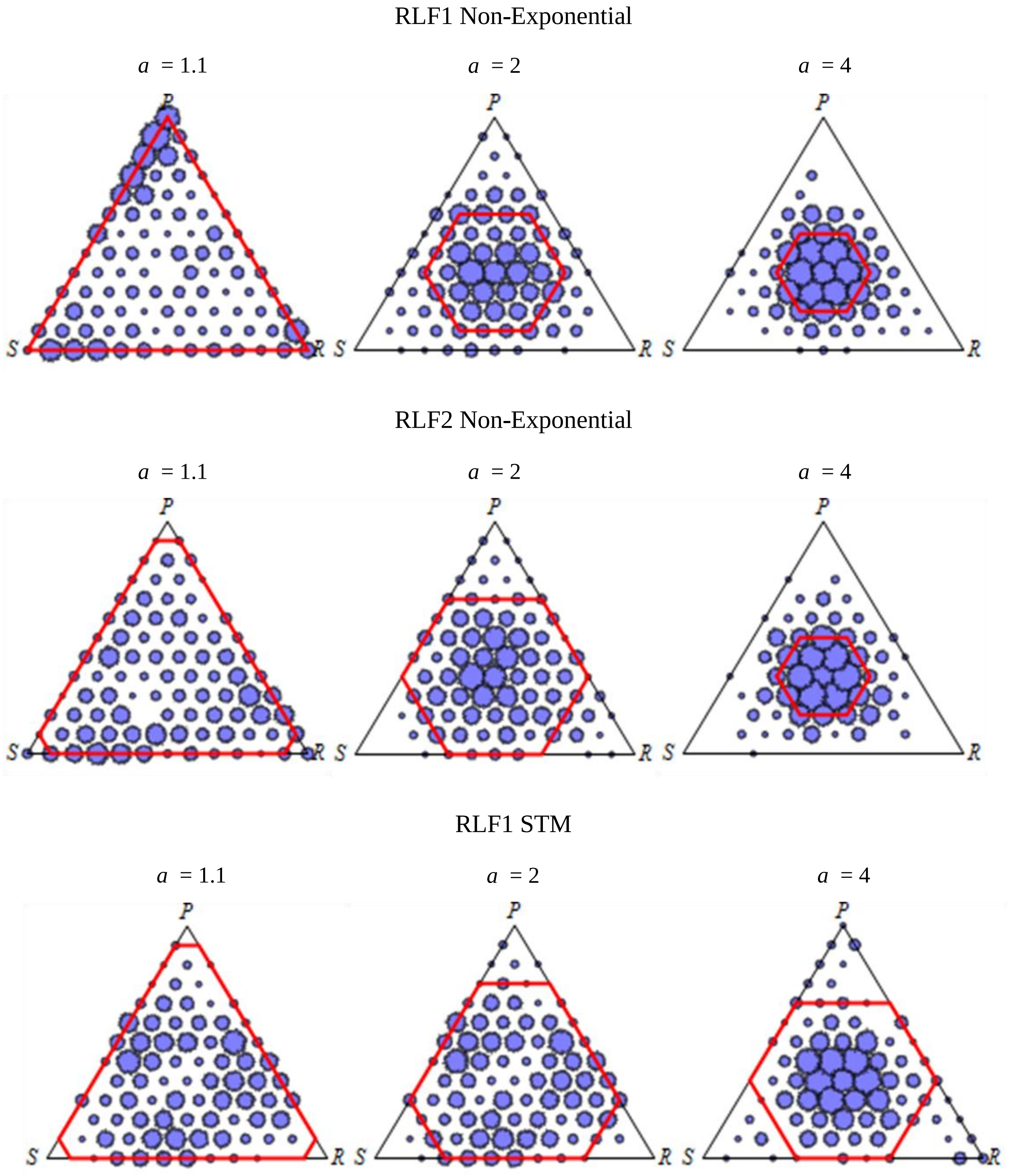

Figure S7. Rock Paper Scissors Configurations in 3 Additional Simulations. The red lines connect the lattice points that are equidistant from the center and cover at least 90% of the data points.

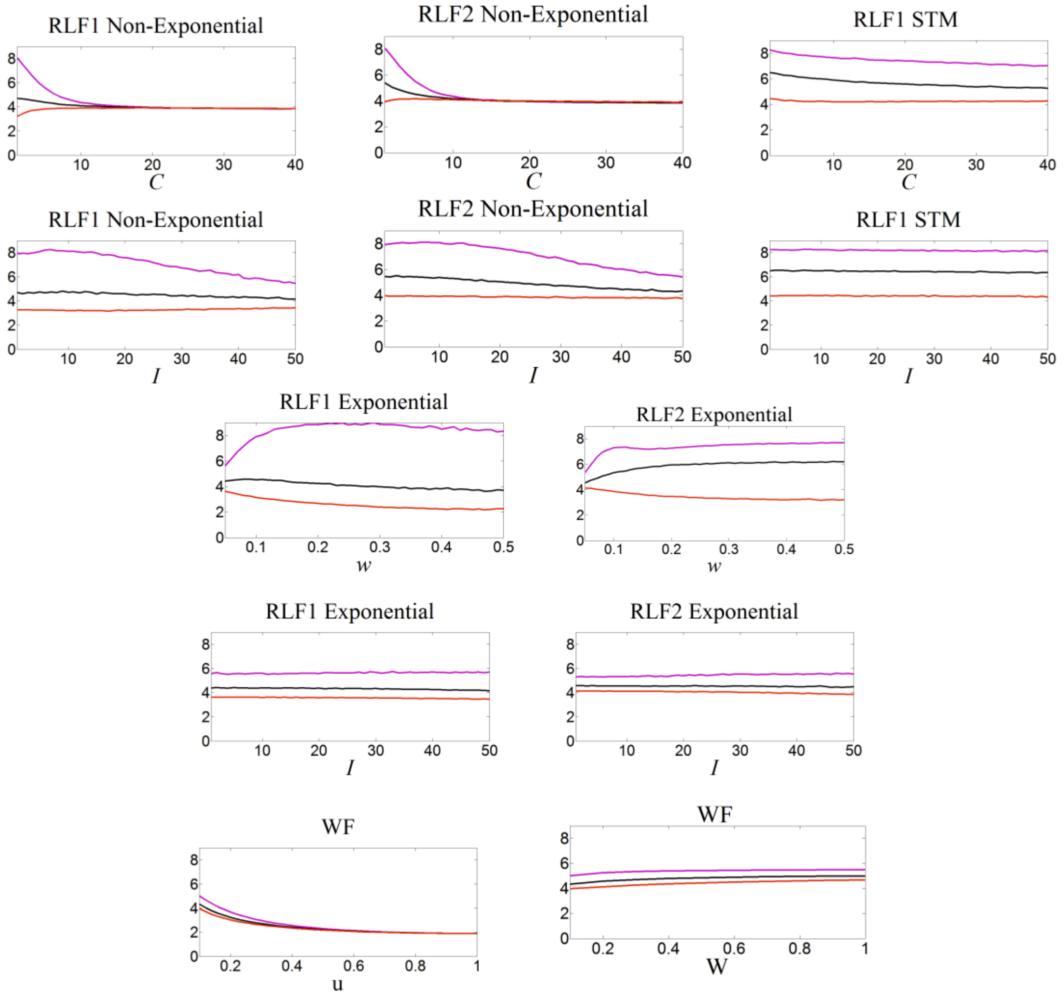

Figure S8. Average Distance for *a* = 1.1 (purple), 2 (black), and 4 (red) for all 6 Simulations depending on Parameter Values.

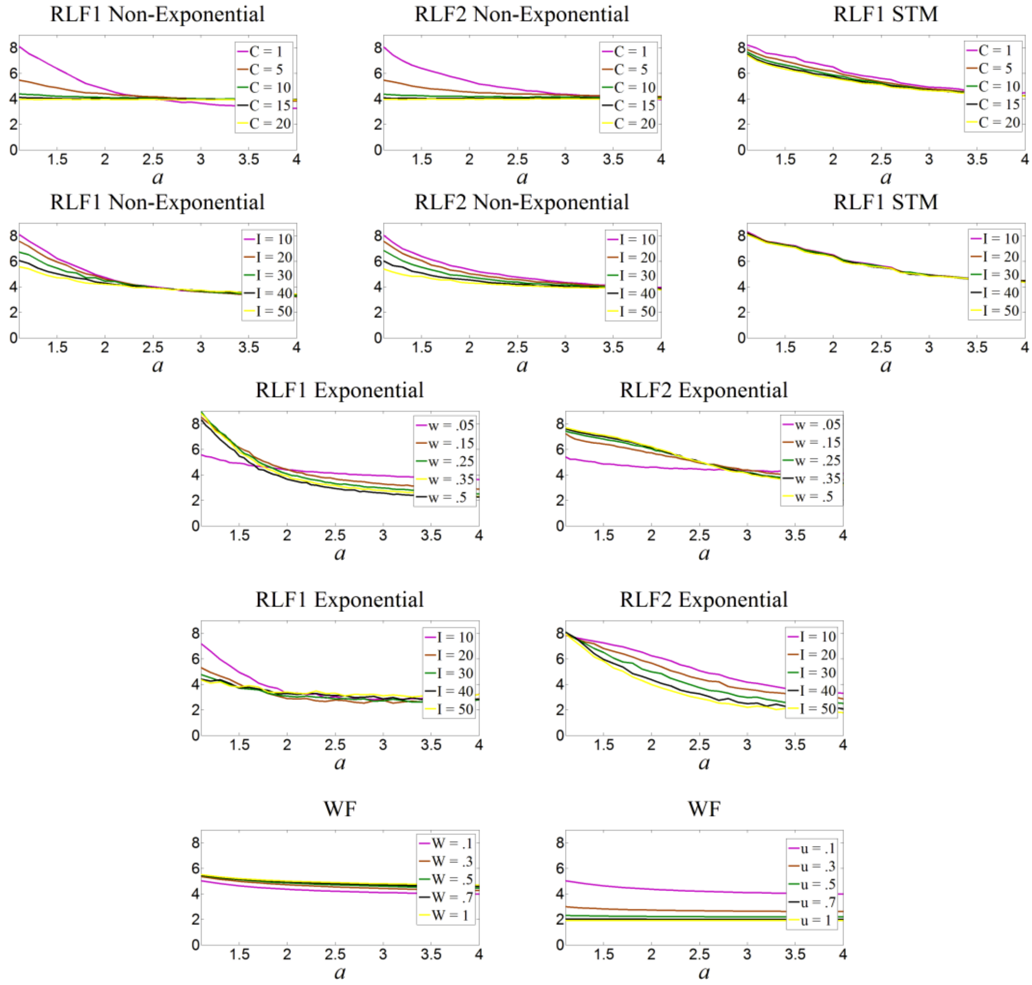

Figure S9. Distance for all 6 Simulations depending on *a*.

References

1. Fischbacher, U. z-Tree: Zurich toolbox for ready-made economic experiments. *Exp. Econ*. **10**, 171-178 (2007).

2. Erev, I., Roth, A.E., Predicting how people play games: Reinforcement learning in experimental games with unique, mixed strategy equilibria. *Am. Econ. Rev*. **88**, 848-881 (1998).

3. Fisher, R.A. *The Genetical Theory of Natural Selection*. (Oxford University Press, 1930).

4. Wright, S. Evolution in Mendelian population. *Genetics* **16**, 97-159 (1931).
